# Polypharmacy and Malnutrition Management of Elderly Perioperative Patients with Cancer: A Systematic Review

**DOI:** 10.3390/nu13061961

**Published:** 2021-06-07

**Authors:** Eiji Kose, Hidetaka Wakabayashi, Nobuhiro Yasuno

**Affiliations:** 1 Department of Pharmacy, Teikyo University School of Medicine University Hospital, 2-11-1 Kaga, Itabashi City, Tokyo 173-8606, Japan; yasuno@med.teikyo-u.ac.jp; 2 Department of Rehabilitation Medicine, Tokyo Women’s Medical University Hospital, 8-1 Kawadacho, Shinjuku City, Tokyo 162-8666, Japan; noventurenoglory@gmail.com; 3 Laboratory of Hospital Pharmacy, School of Pharmacy, Teikyo University, 2-11-1 Kaga, Itabashi City, Tokyo 173-8605, Japan

**Keywords:** cancer treatment, malnutrition, medication, polypharmacy, nutritional management

## Abstract

Malnutrition, which commonly occurs in perioperative patients with cancer, leads to decreased muscle mass, hypoalbuminemia, and edema, thereby increasing the patient’s risk of various complications. Thus, the nutritional management of perioperative patients with cancer should be focused on to ensure that surgical treatment is safe and effective, postoperative complications are prevented, and mortality is reduced. Pathophysiological and drug-induced factors in elderly patients with cancer are associated with the risk of developing malnutrition. Pathophysiological factors include the effects of tumors, cachexia, and anorexia of aging. Metabolic changes, such as inflammation, excess catabolism, and anabolic resistance in patients with tumor-induced cancer alter the body’s ability to use essential nutrients. Drug-induced factors include the side effects of anticancer drugs and polypharmacy. Drug–drug, drug–disease, drug–nutrient, and drug–food interactions can significantly affect the patient’s nutritional status. Furthermore, malnutrition may affect pharmacokinetics and pharmacodynamics, potentiate drug effects, and cause side effects. This review outlines polypharmacy and malnutrition, the impact of malnutrition on drug efficacy, drug–nutrient and drug–food interactions, and intervention effects on polypharmacy or cancer cachexia in elderly perioperative patients with cancer.

## 1. Introduction

Cancers are among the leading causes of morbidity and mortality worldwide, and the number of new cases is expected to rise significantly over the next few decades. At the same time, all types of cancer treatment, such as surgery, radiation therapy, and pharmacological therapies, are improving in sophistication, precision, and in the power to target specific characteristics of individual cancers. All of these treatments, however, are impeded by the frequent development of malnutrition and metabolic derangements in cancer patients, induced by the tumor or by its treatment [1]. It was previously suggested that malnutrition increases the risk of cancer patient mortality and the length of hospital stay [2,3,4,5,6]. Therefore, nutritional management of cancer patients is extremely important.

The relationship between polypharmacy and malnutrition is based on several mechanisms. The long-term use of multiple drugs results in anorexia, generally as a minor or more serious impairment of the digestive tract. Additionally, many drugs have the potential to negatively affect nutritional status by altering the sensory perception of taste, intestinal absorption, and metabolism or inducing the elimination of essential vitamins and minerals [7,8]. Conversely, malnutrition often decreases the bioavailability of drugs and alters their pharmacokinetic and pharmacodynamic properties, which increases the patient’s sensitivity to the drug; even at the usual dose, the drug will have a stronger effect and higher incidence rate of side effects. This gives rise to a vicious circle, wherein polypharmacy, particularly in excess, degrades the nutritional status, and the degraded nutritional status demands increased doses of drugs with the increased occurrence of undesirable side effects [9]. Hence, nutritional status needs to be assessed before prescribing medications.

Elderly patients with cancer often take a large number of medications to prevent or reduce side effects in addition to their multimorbidity. Therefore, they are prone to polypharmacy. As mentioned above, polypharmacy is associated with malnutrition. In elderly perioperative patients with cancer, a multidisciplinary team comprising physicians, pharmacists, nurses, dietitians, and other professionals should be aware of the potential effects of individual drugs and polypharmacy on perioperative nutritional status and seek to reduce negative impacts. Polypharmacy has the potential for adverse clinical outcomes, and it is therefore necessary to synthesize the current evidence to provide a practical direction for future research and clinical practice. This review outlines polypharmacy and malnutrition, the impact of malnutrition on drug efficacy, and drug–nutrient interactions in elderly patients with cancer during the perioperative period.

## 2. Polypharmacy in Elderly Patients with Cancer

Elderly patients with cancer have a higher risk of requiring polypharmacy than patients of the same age without cancer [10]. Most cancer treatments, such as chemotherapy and supportive care regimens, involve the prescription of multiple medications. Furthermore, drugs with anticancer agents are associated with numerous adverse drug reactions, ranging from mild nausea to myelosuppression, which may prompt polypharmacy [11]. Due to these factors, older adults with cancer are at a high risk of requiring polypharmacy. It is estimated that >50% of older patients with cancer are administered at least five medications and that the drug–drug interactions are associated with impaired physical function [12,13].

There are various definitions of polypharmacy, which makes it challenging to understand the scope and impact of the associated problems. A previous review suggested that there are 24 distinct definitions of polypharmacy in general use [14], including concepts ranging from unnecessary or inappropriate medication use to the use of an excessive number of medications [15]. The most common definition of polypharmacy is receiving ≥5 medications daily; however, due to the wide variance in definitions, the appropriateness of medication usage is described only in a minority of the definitions [16]. In Japan, there is no strict definition of how many drugs constitute polypharmacy. The Ministry of Health, Labor and Welfare’s definition of polypharmacy refers to not only the intake of multiple drugs but also to the fact that polypharmacy is associated with conditions that increase the risk of adverse drug reactions, drug errors, and decreased medication adherence. In some cases, adverse drug events may occur when the patient is only administered two or three drugs, as it is equally important to consider the ingredients of the prescription when managing drug–drug interactions. Thus, in defining polypharmacy, the administration of prescriptions must be optimized with the primary aim of ensuring safety rather than focusing only on a uniform number of drugs or types of drugs.

Polypharmacy can lead to various adverse events. Polypharmacy has been associated with increased falls [17], hospitalization [18], decreased physical and cognitive capability [19], impaired activities of daily living (ADL) [20,21], and mortality [22] in older adults without cancer. Hence, elderly patients with cancer are at a greater risk of medication-related events, as they are usually prescribed an extensive number of medicines, both to treat the disease itself and to provide supportive care in treating the side effects of both the disease and the medications used. Therefore, cancer-related therapy contributes to the prevalence of polypharmacy, which can lead to compromised cancer management plans (i.e., postoperative complications, treatment delays, and/or premature treatment discontinuation) [23]. Additionally, in an oncology setting, polypharmacy with inappropriate medications is likely to contribute to the patient’s worsened condition, frailty syndrome, poor physical function, poor survival, and a higher number of comorbidities [24,25]. As is evident from the above descriptions, polypharmacy in elderly patients with cancer is a crucial factor to consider when treating the health conditions of elderly patients with cancer, as it affects the process and outcomes of cancer treatment.

## 3. Effects of Polypharmacy and Malnutrition on Elderly Perioperative Patients with Cancer

Drugs are also associated with causes of malnutrition. Factors in drug-induced malnutrition are the side effects of anticancer drugs. The common side effects of cytotoxic chemotherapy include anorexia, nausea, and vomiting, which directly limit food intake. Additionally, cytotoxic anticancer drugs such as oxaliplatin, cisplatin, doxorubicin, 5-fluorouracil, and irinotecan are taken up by muscle cells. These drugs suppress protein synthesis and induce atrophy, oxidative damage, cellular energy depletion, and apoptotic or necrotic cell death [26]. Decreased de novo lipogenesis and increased lipolysis are additional effects that cisplatin and doxorubicin induce in the adipose tissue [26]. Thus, anticancer drugs affect the nutritional status. Additionally, since many drugs are often prescribed to prevent the side effects of anticancer drugs, elderly perioperative patients with cancer are prone to polypharmacy.

Polypharmacy and anticholinergic drugs have also been associated with the risk of developing malnutrition [27,28,29]. The long-term use of multiple drugs results in anorexia, which generally manifests clinically because of a minor or serious impairment of the digestive tract, which may result in lower food intake and affect the patient’s nutritional status. The inverse relationship between medication use and nutritional status has been demonstrated previously, with 50% of those taking ≥10 medications found to be malnourished or at risk of malnourishment [30]. Elderly perioperative patients with cancer commonly have concomitant lifestyle-related diseases such as hypertension, diabetes mellitus, and atherosclerosis. Drugs used to treat these diseases are also known to affect the nutritional status (see Table 1).

Anticholinergic drugs have also been associated with malnutrition. Elderly patients with cancer often take antipsychotic drugs to prevent delirium and improve symptoms of restlessness. Antipsychotic drugs with anticholinergic effects, such as chlorpromazine, haloperidol, and risperidone, block dopaminergic neurons and potentially inhibit the swallowing reflex, which can induce aspiration pneumonia. Additionally, these drugs can cause extrapyramidal disorders, difficulty opening and closing the mouth, and limitations in lingual movement, thus making mastication, the formation of a food bolus, and its passage into the pharynx difficult.

Malnutrition in patients with cancer is based upon multiple factors. As evidenced from the above descriptions, it is difficult to distinguish between drug-induced malnutrition and malnutrition due to disease or other causes. A multidisciplinary team comprising physicians, pharmacists, nurses, dietitians, and other professionals should comprehensively evaluate malnutrition.

## 4. Effects of Hypoalbuminemia on Drug Efficacy

Elderly patients with cancer may suffer from hypoalbuminemia due to deteriorated swallowing function, loss of dental occlusion, decreased food intake, and the debilitating effects of chronic inflammatory diseases, such as cachexia. When dietary intake is no longer possible and protein and amino acids are not taken into the body as nutrients, the total amount of protein synthesized by the liver reduces, resulting in hypoalbuminemia. If the required amount of energy-producing nutrients is not supplied, amino acids will be used as an energy source and not for body protein synthesis. When protein intake is insufficient, skeletal muscle is broken down and used to sustain life. Hence, when fasting reduces the ability to masticate and swallow, complete loss of the ability to swallow is likely to occur [31]. Additionally, acute inflammation, such as infection or invasion, causes an increase in the serum levels of C-reactive protein. At the same time, albumin synthesis is inhibited, which results in a decrease in the serum levels of albumin. In chronic inflammation, such as cachexia, inflammatory cytokines inhibit albumin synthesis in hepatocytes and promote C-reactive protein production. However, the inhibition of albumin synthesis is usually mild compared with that in acute inflammation.

**Figure 1:**
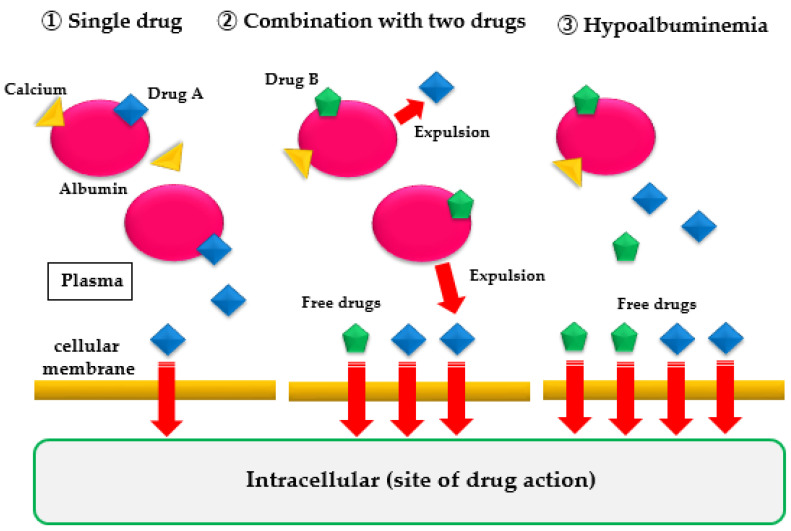
When a single drug is used alone or in combination with two drugs, or in case of hypoalbuminemia, the amount of free drug that does not bind to plasma albumin increases. Consequently, the amount of drug that passes through the cell membrane increases, and the drug effect is strongly expressed.

Drugs sensitive to protein binding affect drug efficacy in hypoalbuminemia. When an administered drug is distributed in the bloodstream, it binds to albumin because of the drug’s pH, electrical charge, steric structure of the molecule, and hydrophilic/hydrophobic properties. Albumin-bound drug molecules are unable to express drug effects. Only free drugs that are not bound to albumin pass through the cell membrane and enter the cell to produce a drug effect. The percentage of drugs that produce a drug effect depends on the type of drug and the amount of albumin and water in the blood. Drug dosage is established by administering the drug to healthy subjects or patients during a clinical trial (dose-finding study). However, there are few data on the appropriate dose for patients with malnutrition and hypoalbuminemia. When albumin in the blood decreases because of undernutrition, the free drugs, which cannot bind to albumin, increase in the blood, resulting in stronger drug effects and more frequent side effects, even at normal doses (Figure 1). Additionally, when the plasma water content is reduced and the blood concentration is increased because of dehydration, the incidence and severity of side effects increase as well. Accordingly, when administering drugs during hypoalbuminemia or dehydration, attention should be given to the changes in the patient’s symptoms.

Albumin binds to calcium, and the binding is affected by pH and temperature. Approximately 40% of the total blood calcium is bound to plasma proteins, primarily albumin. The remaining 60% includes ionized calcium plus calcium complexed with phosphate and citrate. Total calcium (i.e., protein-bound, complexed, and ionized calcium) is usually determined by clinical laboratory measurement. However, ideally, ionized (or free) calcium should be estimated or measured because it is the physiologically active form of calcium in plasma and because its blood level does not always correlate with total serum calcium.

## 5. Interaction of Drugs with Nutrients or Diet

### 5.1. Effects of Drugs on Nutrients

More than 250 drugs have been reported to have adverse effects on the patient’s nutritional status because of drug-induced alterations in taste, intestinal absorption, and metabolism or excretion of essential vitamins and minerals [7,8]. Table 1 shows the effects of major drugs on nutrients.

#### 5.1.1. Antihypertensive Drugs and Zinc

Antihypertensive drugs such as thiazide diuretics, angiotensin receptor blockers, angiotensin-converting enzyme inhibitors, and potassium-conserving diuretics decrease zinc levels [7]. Zinc deficiency is a common cause of taste disorders, which can lead to weight loss and malnutrition. Thus, zinc administration may be used to improve or prevent these symptoms. A Cochrane review that examined the improvement of taste perception due to zinc supplementation in patients with idiopathic and zinc-deficient taste disorders found very low-quality evidence that zinc supplementation improves taste perception (relative risk: 1.42, 95% confidence interval: 1.09–1.84; 292 participants, two trials) [32]. Zinc could be useful in the prevention of oral toxicities during irradiation; however, it does not alleviate chemotherapy-induced side effects [33]. Accordingly, several studies have proposed that zinc does not have a positive effect on patient weight or food intake [32].

#### 5.1.2. Acetylcholinesterase Inhibitors

The typical side effects of acetylcholinesterase inhibitors include nausea, vomiting, diarrhea, and anorexia, and each of these symptoms may lead to weight loss. Weight loss is often observed after 3 months of use, but studies have shown that weight loss does not persist over the long-term use of the inhibitors [34]. However, its use in elderly patients with weakness or anorexia should be carefully considered [34].

#### 5.1.3. Proton Pump Inhibitors (PPIs)

Several studies have examined the association between long-term PPI use and the risk of developing vitamin B_12_ deficiency [35,36]; most, [36] but not all [37], studies reported a 2- to 4-fold increased risk of vitamin B_12_ deficiency associated with PPI therapy. The complex relationship between PPI use and nutritional status has not been fully elucidated. However, it has been reported that the long-term use of PPIs may improve nutritional status in elderly patients admitted to a long-term care ward, convalescent rehabilitation ward, or community-integrated care ward [35].

Several meta-analyses have reported that PPI use is also associated with the development of hypomagnesemia [38,39]. A dose–response relationship was found between PPI use and the development of hypomagnesemia [38]. Hypomagnesemia increases the risk of developing cardiovascular events [40]. Thus, PPI users should be aware of the risk of developing hypomagnesemia.

PPIs can decrease the absorption of water-insoluble calcium (e.g., calcium carbonate) [41]; however, this effect is not relevant for water-soluble calcium salts [42] or calcium-containing milk or cheese [43]. When calcium supplementation is necessary for patients taking PPIs, calcium supplements that do not require acid for absorption, such as calcium citrate, are recommended. PPI-induced hypochlorhydria can augment osteoclastic activity, thereby decreasing bone density [44,45]. Hence, calcium supplementation is necessary for such patients. Although an association between PPI use and bone fracture is plausible, a causal link has not been established [46].

Gastric acid plays a role in the absorption of nonheme iron, and the use of PPIs has been associated with decreased iron absorption [47,48,49,50,51]. However, few studies have specifically evaluated the potential association between PPIs and iron deficiency [52]. In most cases, the decreased absorption of iron does is not clinically significant [52]. In patients with Zollinger–Ellison syndrome, 6 years of taking PPIs was not associated with decreased total body iron levels or iron deficiency [53]. Conversely, PPI use in patients with hereditary hemochromatosis was associated with a significant reduction in the absorption of nonheme iron over the short term as well as a significant reduction in annual phlebotomy requirements over the long term [48]. Such patients may need a higher dose or a longer duration of supplementation.

#### 5.1.4. Statins

Statins decrease the production of coenzyme Q_10_ (CoQ_10_) by inhibiting mitochondrial oxidative phosphorylation and inducing mitochondrial apoptosis [7,54]. Decreased CoQ_10_ results in decreased adenosine triphosphate production and energy deficiency. Consequently, the number of cellular processes decreases, which may induce frailty and sarcopenia.

Mitochondrial function has been associated with myopathy, which is a side effect of taking statins. Since skeletal muscle is highly energy-consuming and deeply dependent on mitochondrial activity, mitochondrial dysfunction is largely associated with the development of statin-induced myopathy [54]. In a recent meta-analysis examining the effects of CoQ_10_ on statin-induced myopathy, the concomitant use of CoQ_10_ significantly improved statin-related muscle symptoms, such as muscle pain, muscle cramps, and muscle fatigue [55]. A previous study demonstrated a reduction in CoQ_10_ after statin treatment, which may have been associated with statin-induced myopathy [56]. As is evident from the above description, the concomitant use of statin with CoQ_10_ supplements may be a complementary approach to symptom relief of statin-induced myopathy.

#### 5.1.5. Aspirin

The long-term use of high-dose aspirin has been associated with decreased vitamin C levels. It has been suggested that this leads to gastritis, peptic ulcer disease, nausea, anorexia, and thinning of the gastric mucosa with hypotrophy [7]. At this time, there is no evidence suggesting a decrease in vitamin C levels or the need for vitamin C supplementation in patients taking low-dose aspirin for primary or secondary prevention of cardiovascular disease.

#### 5.1.6. Metformin

Metformin causes vitamin B_12_ deficiency in a dose- and duration-dependent manner [7]. Vitamin B_12_ deficiency is associated with serious outcomes, such as anemia and cognitive impairment; thus, patients taking metformin should have their vitamin B_12_ levels measured regularly and consider supplementation if they are deficient.

Metformin inhibits the breakdown of muscle proteins and affects muscle mass and strength. Metformin activates 5′ adenosine monophosphate-activated protein kinase, suppresses inflammatory responses, and inhibits muscle protein degradation. Studies of sarcopenia have revealed numerous health benefits of 5′ adenosine monophosphate-activated protein kinase activation. First, sarcopenia promotes skeletal muscle protein synthesis and cell proliferation. Second, sarcopenia inhibits apoptosis in skeletal muscle. Third, sarcopenia improves dysfunction by promoting mitochondrial biogenesis. Finally, sarcopenia induces the growth of bone marrow-derived muscle progenitor cells in skeletal muscle [57]. Specifically, in a double-blind randomized controlled trial, metformin was found to improve the walking speed of older adults with no diabetes, which indicates that metformin has a positive effect on lower limb muscle strength [58]. In a prospective cohort study of elderly women with diabetes, women who took metformin experienced reduced loss of walking speed compared to the controls [59]. In another cohort study, metformin reduced the age-related loss of lean body mass in elderly male diabetics [60]. These findings suggest that metformin has a positive effect on muscle mass and muscle strength.

#### 5.1.7. Sodium Glucose Transporter 2 Inhibitors

Sodium glucose transporter 2 (SGLT-2) inhibitors may induce sarcopenia by causing protein breakdown, particularly in elderly patients with inadequate dietary intake. SGLT-2 inhibitors cause a decrease in serum insulin levels and an increase in glucagon levels. Consequently, they reduce the uptake of glucose and amino acids into muscle and promote protein breakdown [61]. Dapagliflozin, ipragliflozin, and empagliflozin have been shown to decrease muscle mass and skeletal muscle mass index [62,63,64,65]. However, further studies are needed to determine the frequency of the occurrence of SGLT-2 inhibitor-related sarcopenia and whether SGLT-2 inhibitors cause diabetes-related sarcopenia, as clinical data are limited.

**Table 1:** Effects of drugs on nutrients.

Drugs	Effects of Drugs on Nutrients	Symptoms Caused	Countermeasure	References
Antihypertensive drugs: thiazide diuretics, ARBs, ACE inhibitors, and potassium-retaining diuretics	Zinc deficiency	Taste disorder, anorexia, lethargy, and delayed wound healing	•Determination of zinc levels in plasma or urine •Blood pressure monitoring to determine the need for continued administration	[7]
Acetylcholinesterase inhibitors	Unknown	Nausea, vomiting, diarrhea, and loss of appetite	•Monitoring changes in appetite and weight loss •Assessing the benefits of using medications for the risk of malnutrition	[34]
Proton pump inhibitors	Deficiency of VB_12_, Mg, Ca, and Fe	Clostridium difficile diarrhea, pneumonia, femoral neck fracture, hypomagnesemia, and hypocalcemia	•Measurement of VB_12_, Mg, Ca, ferritin, and FRAX score •Evaluate the need for continued administration	[35,36,37,38,39,41,53]
HMG-CoA reductase inhibitors (stains)	CoQ_10_ deficiency	Frailty, sarcopenia, and myopathy	•Examining the use of CoQ_10_ in combination •Evaluate the need for continuous administration in patients over ≥75 years	[7,54]
Long-term, high-dose aspirin	VC deficiency	Gastric mucosal thinning	•Long-term use of low-dose aspirin (80–400 mg/day) •VC supplementation if higher doses are needed	[7]
Metformin	•VB_12_ deficiency •Inhibits the breakdown of muscle proteins	•Anemia, fatigue, and cognitive impairment •Improvement of muscle mass and strength	Monitor vitamin B_12_ and consider switching to another drug if it is low	[7]
SGLT-2 inhibitors	Protein degradation	Sarcopenia, decrease in muscle mass, and skeletal muscle mass index	Consider the need for continued administration	[62,63,64,65]
Diuretics (loop, thiazide, and osmotic), corticosteroids, kanzo, insulin, β_2_-adrenergic stimulation	Lower potassium	Vomiting, anorexia, weakness, muscle weakness, tetany	Monitor potassium and consider eating foods rich in potassium or taking supplements	[66,67,68,69,70]

Abbreviations: Angiotensin-converting enzyme inhibitors, ACE inhibitors; angiotensin II receptor blockers, ARBs; calcium, Ca; coenzyme Q_10_, CoQ_10_; hydroxymethylglutaryl-CoA, HMG-CoA; ferrum, Fe; fracture risk assessment tool, FRAX; magnesium, Mg; sodium glucose transporter-2, SGLT-2; vitamin B_12_, VB_12_; vitamin C, VC.

#### 5.1.8. Diuretics, Corticosteroids, Kanzo (Kampo), Insulin, and β_2_-Adrenergic Stimulation

Loop, thiazides, and osmotic diuretics cause hypokalemia [66]. These drugs act on the renal tubules to inhibit the reabsorption of water along with sodium, thereby producing a diuretic effect. At the same time, they promote the excretion of potassium, resulting in hypokalemia. Hypokalemia may be accompanied by symptoms such as vomiting, loss of appetite, and weakness. The diuretic effect also causes dry mouth. These symptoms may affect food intake. Steroids cause hypokalemia via their mineralocorticoid action [67]. The main ingredient in kanzo is glycyrrhizic acid. Glycyrrhizic acid acts on the distal tubules of the kidneys, causing retention of sodium and water in the body, hypokalemia, and increased blood pressure [68]. These effects are referred to as pseudohypoaldosteronism. Both insulin and β_2_-adrenergic stimulation activate potassium uptake by stimulating activity of the adenosine triphosphatase sodium/potassium pump predominantly in skeletal muscle [69,70].

### 5.2. Nutrient–Drug and Diet–Drug Interactions

Typical nutrients or diets, including vitamins, calcium, high-fat meals (approximately ≥900–1000 kcal), and high-protein meals (protein accounting for ≥20% of the total caloric content of the meal), and their drug interactions are listed in Table 2. It is important to be aware of these interactions and to keep their risks in mind when managing patient treatment plans. However, nutrient–drug or diet–drug interactions are still in the developmental stage, and there are not many cases in which the management of such interactions has been established. Under these circumstances, careful observation in the clinical setting is essential to assess the frequency and severity of adverse effects due to nutrient–drug or diet–drug interactions and to identify unknown interactions.

## 6. Drug and Eating Habits

Changes in eating habits also affect the efficacy of drugs. A previous study has examined the relationship between the time trends of caloric intake and statin use. Caloric and fat intakes increased among statin users over time, which was not true for nonusers. The increase in BMI was faster for statin users than for nonusers. Efforts aimed at dietary control among statin users may be becoming less intensive [103]. Additionally, another study has shown that eating habits affect blood pressure control in outpatients treated with antihypertensive drugs. Habitual intake of foods rich in potassium and magnesium were associated with reduced intensity and cost of medication and with preservation of blood pressure control in elderly hypertensive outpatients [104]. This change in eating habits will also affect the efficacy of the drug. Elderly perioperative patients with cancer often have concomitant lifestyle-related diseases. It should be noted that a change in eating habits may also have a small effect on nutritional status.

## 7. Intervention Effects on Polypharmacy, Cancer Cachexia, and Rehabilitation Nutrition

### 7.1. Intervention Effects on Polypharmacy

Effective interventions for polypharmacy have been studied. A systematic review by Hill-Taylor et al. revealed that intervention using the STOPP&START criteria [105] reduced the proportion of potentially inappropriate medications (PIMs) prescribed. PIMs include prescriptions of an incorrect dose, frequency, or mode of administration or duration that are likely to result in clinically significant drug–drug or drug–disease interactions or have no clear evidence-based clinical indication [106,107]. Another intervention study showed that hospital pharmacists reduced the number of PIMs by using the STOPP&START criteria [108]. In this study, out of 651 PIMs, 292 (44.9%) were changed or discontinued [109]. A retrospective cohort study of 569 older adults based on “rehabilitation pharmacotherapy” [110] reported an association between a decrease in the Beers criteria for [111] PIMs and an improvement in the motor ADL at discharge [112].

Alternatively, a recent systematic review of interventions to reduce polypharmacy failed to show a benefit based on clinical evidence such as mortality rates, the number of hospitalizations, and the frequency of falls [113,114,115]. In patients admitted to an acute care ward, the frequency of emergency room visits and readmissions was smaller in the multimodal intervention group, which combined a medication reduction review with motivational interviewing and follow-up by a multidisciplinary team, than in the other group with usual care [116]. However, there was no significant difference in the outcomes between the usual care group and the medication review-only group. Similarly, interventions that combined patient interviews and patient education with a medication review reduced the number of hospital visits and drug-related hospitalizations [117] and the frequency of emergency department visits [117,118]. Thus, a reduction of polypharmacy is expected to improve the frequency of emergency department visits, readmissions, and quality of life. However, if the goal is to improve clinical outcomes such as mortality rates, the number of hospitalizations, and the frequency of falls, patient-centered multimodal interventions such as the combination of a medication review, multidisciplinary collaboration, and patient education may be more effective.

### 7.2. Intervention Effect on Cancer Cachexia

Cachexia includes “objective” components (e.g., inadequate food intake, weight loss, inactivity, loss of muscle mass, and metabolic derangements inducing catabolism) and “subjective” components (e.g., anorexia, early satiety, taste alterations, chronic nausea, distress, fatigue, and loss of concentration) [119]. Thus, comprehensive treatment requires a multitargeted and multidisciplinary approach, such as nutrition, rehabilitation, and pharmacotherapy aimed at evaluating objective signs and symptom relief.

The European Society of Medical Oncology Clinical Practice Guidelines for cancer cachexia indicated that nutritional interventions for cancer cachexia will meet energy and nutrient requirements and simultaneously normalize metabolic status, including strength training and reduction of systemic inflammation and pain relief. To maintain nutritional status, at least 25–30 kcal/kg/day and at least 1.2 g protein/kg/day is recommended, with adjustments made to the regimen as required. Furthermore, regimens with fat accounting for half of the nonprotein calories are recommended [120]. The American Society of Clinical Oncology guideline for cancer cachexia indicated that the only nutritional intervention recommended was nutritional counseling by a registered dietitian. A high-protein, high-energy, nutrient-dense diet is the recommended dietary guideline [121].

There are a limited number of drugs that can be used in the pharmacotherapy of cancer cachexia. The European Society of Medical Oncology guidelines state that short-term corticosteroids and progestins can be used to increase appetite and weight gain [120]. The use of olanzapine may also be considered for the treatment of appetite and nausea in patients with advanced cancer. The American Society of Clinical Oncology guidelines similarly recommended the use of progesterone derivatives and corticosteroids for appetite improvement and weight gain [121]. Anamorelin was approved in Japan in January 2021 for the first time in the world for cancer cachexia in patients with non-small-cell lung cancer, gastric cancer, pancreatic cancer, or colorectal cancer. However, it has not been approved in Europe based on the findings from the ROMANO studies [122,123], which did not show the improvement in muscle strength that was demonstrated by the Japanese trials. The approval of the use of anamorelin is expected to change the clinical practice of cancer cachexia in Japan [124].

To reverse cancer cachexia, lean body mass should be increased, and muscle function must be restored. Hence, a combination of improved nutrition, physical exercise that can improve muscle function, and pharmacotherapy may be an option for reversing cachexia in patients with cancer.

### 7.3. Intervention Effect on Rehabilitation Nutrition

The concept of rehabilitation nutrition, which combines rehabilitation and nutritional therapy, has attracted attention in recent years [125]. Rehabilitation nutrition includes holistic assessment by the International Classification of Functioning, Disability, and Health; assessment for nutritional disorders, sarcopenia, and excessive or inadequate intake of nutrients; diagnosis of rehabilitation nutrition; and goal setting. Rehabilitation nutrition improves the nutritional status of sarcopenia patients with disabilities and frail, older people, besides maximizing their functions, activities, participation, and quality of life. This is achieved via “nutrition care management in consideration of rehabilitation” and “rehabilitation in consideration of nutrition” [125]. Rehabilitation nutrition practices based on the Rehabilitation Nutrition Care Process can improve function and ADL in cases of malnutrition and sarcopenia [126]. The clinical practice guidelines of the 2020 rehabilitation nutrition edition weakly recommended the use of enhanced nutritional therapy in cerebrovascular disease, hip fracture, cancer, and acute disease [127]. Furthermore, improving the nutritional status can recover swallowing ability and ADL in malnourished patients. Malnourished patients with stroke, hip fracture, or pneumonia are more likely to achieve improved swallowing function and ADL following nutrition improvement [128,129,130]. Therefore, in the case of malnutrition and sarcopenia, rehabilitation should be combined with aggressive nutritional therapy so that energy and protein are added to the daily energy expenditure, which will improve the nutritional status.

## 8. Conclusions

Pathophysiological and drug-induced factors are associated with the risk of developing malnutrition in elderly patients with cancer. Polypharmacy is associated with malnutrition. The long-term use of several drugs, including anticancer drugs, leads to anorexia as a gastrointestinal disorder and induces malnutrition. Furthermore, several drugs are known to interact with nutrients and diet, which may adversely affect the patient’s nutritional status. Conversely, malnutrition may affect pharmacokinetics and pharmacodynamics, potentiate drug effects, and induce or worsen side effects. Therefore, nutritional management of elderly patients with cancer during their perioperative period should consider pathophysiological factors such as tumors or cancer cachexia as well as drug-related factors. In other words, it is necessary to simultaneously evaluate the development of malnutrition from the perspective of drug interactions and the development of adverse drug events from the perspective of malnutrition. To this end, multidisciplinary teams should put forth an effort to recognize and mitigate the potential impact of the patient’s perioperative nutritional status.

## Figures and Tables

**Table 2:** Nutrient–drug and diet–drug interactions.

Vitamin			
**Vitamin**	**Drug**	**Effects of Interactions**	**Reference**
A	Paclitaxel	Vitamin A inhibits the metabolism of paclitaxel and increases the blood concentration of paclitaxel	[71]
B_6_	Phenytoin	Decrease in blood phenytoin level	[72]
	Aluminum hydroxide	Decreased absorption of riboflavin and prolonged time for urinary excretion to reach its maximum	[73]
	Levodopa	Accelerates levodopa degradation and reduces its migration in the brain	[74]
B_12_	Cimetidine	Decreased absorption of vitamin B_12_ with intake of 1000 mg/day	[75]
C	Iron sulfate	Iron absorption increases with concurrent intake of ≥200 mg of vitamin C	[76]
D	Thiazide Diuretics	Hypercalcemia	[77]
	Digoxin	Hypercalcemia can lead to digitalis poisoning	[78]
E	Warfarin	Prolongation of prothrombin time and appearance of ecchymosis	[79]
K	Warfarin	Decreased effect of warfarin	[80]
**Calcium**			
**Drug**		**Effects** **of Interactions**	**Reference**
Aspirin		AUC and Cmax significantly decreased by approximately 30% and 28%, respectively, and disappearance speed rate of aspirin also decreased	[81]
Tetracycline antibiotics		Calcium and the drug bind together to form a chelate, which reduces absorption from the small intestine	[82]
New quinolone antibiotics			[83]
Bisphosphonate osteoporosis drugs			[84]
Estramustine phosphate			[85]
Digoxin		Large amounts of calcium should be avoided as hypercalcemia causes digitalis toxicity	[86]
Alfacalcidol, rocartrol, andeldecalcitol		Promotes the absorption of calcium in the intestinal tract	[87]
**High-fat meals**			
**Drug**		**Effects of Interactions**	**Reference**
Cyclosporine		Significantly increased blood concentration (approximate 1.5-fold AUC increase)	[88]
Theophylline		Faster absorption of theophylline, significant increase in AUC	[89]
Griseofulvin		Significantly increased absorption of griseofulvin by approximately 120%	[90]
Oxycodone		Approximately 20% increase in AUC	[91]
Ivermectin		AUC increased to approximately 2.6 times that of fasting administration	[92]
Erlotinib		AUC of erlotinib almost doubled compared to fasting	[93]
Sirolimus		Increased tmax, Cmax, and AUC	[94]
Regorafenib		Decrease in Cmax and AUC of active metabolites	[95]
Sorafenib		Decrease in plasma concentration	[96]
Lenalidomide		Decrease in AUC and Cmax	[97]
Trametinib		Plasma trametinib AUC and Cmax of plasma trametinib were decreased by approximately 10% and 70%, respectively, compared to fasting	[98]
Dabrafenib		AUC and Cmax decreased by approximately 31% and 51%, respectively, compared to fasting	[99]
**High-protein meals**			
**Drug**		**Effects of Interactions**	**Reference**
Propranolol		74% increase in clearance of propranolol	[100]
Theophylline		32% increase in the clearance of theophylline and 26% decrease in half-life	[100]
Levodopa		Reduced levodopa absorption due to drug transporter competition	[101]
Aluminum hydroxide		Decreased antacid effect of aluminum hydroxide	[102]

Abbreviations: Area under the curve, AUC; maximum serum concentration, Cmax.

## Data Availability

Not applicable.
